# Mental health of children with epilepsy in Ukraine during the war

**DOI:** 10.1002/epi.70251

**Published:** 2026-04-21

**Authors:** Volodymyr Kharytonov, J. Helen Cross, Andriy Dubenko, Samuel Wiebe, the ILAE Emergency Task Force for Ukraine

**Affiliations:** ^1^ Kyiv City Children's Clinical Hospital #1 Kyiv Ukraine; ^2^ UCL NIHR BRC Great Ormond Street Institute of Child Health London UK; ^3^ Institute of Neurology, Psychiatry, and Narcology, P.V. Voloshyn of NAMS of Ukraine Kharkiv Ukraine; ^4^ Department of Clinical Neurosciences Cumming School of Medicine, University of Calgary Calgary Canada

**Keywords:** anxiety, conflict, epilepsy in childhood, mental health, post‐traumatic stress disorder

## Abstract

**Objective:**

The ongoing conflict in Ukraine has created a severe humanitarian crisis, disproportionately affecting vulnerable populations such as children with chronic conditions. We set out to determine information about mental health in children with epilepsy in Ukraine affected by the conflict.

**Methods:**

Mental health outcomes in 213 Ukrainian children with epilepsy during the conflict were screened using standardized instruments for post‐traumatic stress disorder (PTSD; Harvard Trauma Questionnaire), anxiety (7 item Generalized Anxiety Disorder module, GAD‐7), depression (Patient Health Questionnaire‐9, PHQ‐9), and epilepsy severity (Global Assessment of Severity of Epilepsy scale, GASE). Data were collected between February and June 2023 through self‐ or proxy‐completed surveys.

**Results:**

Participants (14.6%) screened positive for PTSD symptoms, with girls significantly more affected than boys. A high proportion screened positive for symptoms of anxiety and depression, with 22% in the range for severe anxiety and 55% in the moderate‐to‐severe depression range. Epilepsy severity strongly correlated with mental health burden. Access to care was severely disrupted: 76% reported difficulty obtaining antiseizure medications, and 51% struggled to access medical services, contributing to increased seizure frequency in 56% of cases. Regression analyses identified epilepsy severity and barriers to medical care as key predictors of adverse mental health outcomes.

**Significance:**

These findings underscore the compounded impact of war and chronic illness on children's psychological well‐being and highlight the urgent need for trauma‐informed, multilevel interventions, and improved access to health care for this vulnerable population.

**Key points**

- War significantly impacts mental health and seizure control in children with epilepsy.
- Girls showed higher trauma‐related symptoms than boys.
- Post‐traumatic stress syndrome (PTSD) prevalence was lower than expected, possibly due to proxy reporting or displacement effects.
- Access to medical care and anti‐seizure medications is critical; lack thereof worsens mental health and epilepsy severity.
- Interventions should be trauma‐informed, multilevel, and resilience‐oriented, with targeted screening for mental health in this vulnerable group.

## 1 INTRODUCTION

The ongoing conflict in Ukraine has precipitated a humanitarian crisis of immense proportions, inflicting profound suffering on the civilian population. Among those most vulnerable are children, who constitute a significant portion of those affected by the hostilities. Prior to the full‐scale invasion in 2022, Ukraine was home to ~7.5 million children.^1^ Since then, the war has triggered the displacement of around 5 million children, with roughly half seeking refuge in safer regions within Ukraine and the other half fleeing to other countries.^1^ This mass displacement inherently exposes a vast number of children to traumatic experiences, including loss, violence, and the disruption of their normal lives.^1^ The sheer scale of this displacement suggests a potentially massive mental health crisis, particularly for those with pre‐existing health conditions.^1^

Even before the current conflict, a significant number of children in Ukraine were living with epilepsy, a chronic neurological condition characterized by recurrent seizures. Estimates from before February 2022 indicate that ~40 000 children in Ukraine had epilepsy.^2^ Prevalence studies have shown that around 3.21% of the child population in Ukraine are affected by this condition.^3^ This substantial population of children with epilepsy now faces the added burden of war trauma, creating a situation of compounded vulnerability. The intersection of a pre‐existing neurological disorder with the severe psychological impact of war necessitates a focused examination to understand the unique challenges these children encounter and to inform appropriate support strategies.

A recent study reporting on mental health in adolescents exposed to war in Ukraine, using self‐reported questionnaires, showed they were more likely to screen positive for depression, anxiety, clinically relevant psychological trauma, eating disorders, and substance use disorder than those not exposed to war.^4^ Mental health disorder rates in children, however, are known to be high in epilepsy, and higher than in other chronic conditions, such as diabetes,^5^ in the absence of conflict. A previous survey and screening of an adult population with epilepsy in Ukraine revealed 46% suffered psychological distress, 62% experienced anxiety, 50% were depressed, and 59% rated their epilepsy as somewhat severe or worse than before the conflict.^6^ Therefore, we set out to determine information about mental health in children with epilepsy in Ukraine, aiming to evaluate the prevalence of these mental health conditions in this specific population, explore the factors that exacerbate their vulnerabilities, and identify the particular challenges they have faced in accessing health care, medication, and psychological support.

Ultimately, this analysis seeks to contribute to a deeper understanding of these critical issues for the benefit of children with epilepsy affected by war.

## 2 MATERIALS AND METHODS

The participants of the study were parents and carers of children <10 years of age with a diagnosis of epilepsy, and children with epilepsy ≥10 years of age, who before and during the beginning of the Russian‐Ukrainian war resided in Ukraine. We assessed the mental health well‐being of these children using questionnaires that were available to researchers in Ukraine during the war. These included the Harvard Trauma Questionnaire (HTQ), General Anxiety Disorder‐7 (GAD‐7), and Patient Health Questionnaire (PHQ) ‐9 scales, either completed by the children themselves or by their carers. Each questionnaire had a unique code for each child, so if scores were concerning, the family were contacted and referral/further assessment arranged. Information about use and access to anti‐seizure medications (ASMs) was obtained through direct questions.

The HTQ was used to assess any degree of post‐traumatic stress disorder (PTSD),^7^ specifically, Part IV: Trauma Symptoms Scale, of the original HTQ, Version 1, completed by the child where possible, or otherwise the parent by proxy. Items 1 to 16 of the HTQ map to the Diagnostic and Statistical Manual of Mental Disorders, 4th Edition (DSM‐IV) PTSD symptom clusters, with the following symptom categories: Items 1–4 relate to re‐experiencing, 5–8 to arousal, and 9–15 to avoidance/numbing. Item 16 is noted as a general emotional distress item. The remaining HTQ items aim to assess culture‐specific reactions to violence, displacement, and the psychosocial functioning consequences of complex trauma experienced by refugee populations. The symptom severity scale is reported as a mean score across all answered items, rather than a single summed total score. Therefore, mean scores fall within the 1 to 4 range. A common clinical cutoff score of 2.5 on this mean scale is often used to identify individuals considered symptomatic for PTSD, suggesting a high likelihood of the disorder. The PHQ‐9^8^, ^9^, ^10^ was the first self‐report questionnaire for use in primary care to aid the diagnostic process of depression, using criteria from the DSM‐IV Test Revision (DSM‐IV‐TR).^11^ The 7‐item Generalized Anxiety Disorder module (GAD‐7)^12^ takes less than 3 min to complete and is easy to score. The GAD‐7 has been recommended by the American Psychiatric Association as a useful instrument for measuring the severity of GAD according to the new DSM‐5 criteria.^13^, ^14^ To assess patient‐perceived severity of epilepsy we used the Global Assessment of Severity of Epilepsy (GASE) scale,^15^ a validated, 7‐point, single‐item global rating of severity of epilepsy with response options ranging from 1 = not severe at all to 7 = extremely severe. Although typically a clinician‐report instrument, there is precedent for it being used for self report.^14^, ^16^, ^17^

We acknowledge that some of our scales are best suited for children 11 years of age or older. However, our goal was to capture the full age spectrum of the pediatric population affected by the war, including the adolescent population. Wartime conditions and the availability of validated Ukrainian translations constrained instrument selection. As there were no readily available, translated, and validated instruments for young children, we used these tools to collect proxy responses from caregivers regarding the mental health and quality of life of younger children.

We used a self‐administered, user‐friendly paper‐and‐pencil cross‐sectional survey method, distributed to children with epilepsy and their parents. An electronic form was also created with the same content as the paper version. We accessed health care centers across all regions in Ukraine, spanning from February to June 2023, 1 year into the war. During the questionnaire administration period, many children were relocated from cities to towns and villages. Consequently, the majority of questionnaires were completed independently by children or their parents, either online or via paper versions. Medical supervision was present in fewer than 25%–30% of cases.

### 2.1 Statistical analysis

We used descriptive statistics for the overall data. Parametric and non‐parametric statistics were used to compare normally and non‐normally distributed data, respectively. Bonferroni correction was applied for multiple comparisons. Effect size using Cohen's *d* was used to quantify the magnitude of differences between groups, as this is a common metric that allows for assessment across a broad range of domains. Logistic regression assessed the association between predictor variables and dichotomous outcomes, with results expressed as odds ratios (ORs). Four separate multivariate linear regression models assessed the association of predictor variables on scores of GAD‐7, PHQ‐9, GASE, and HTQ. The predictor variables for all models were age, sex, trouble getting ASMs, trouble getting medical care, interaction between problems accessing ASMs and medical care, changing residence, epilepsy severity (except when predicting GASE), having seizures before the war, and increasing seizure frequency after the war. Mediation analysis was used to further explore factors that mediated selected mental health outcomes. When required for analyses, the scores of mental health instruments were dichotomized using thresholds for levels of severity described in the literature, for example, a score >2.5 on the HTQ indicating a high likelihood of PTSD.

Ethical approval was obtained from the Institute of Neurology, Psychiatry and Narcology of the National Academy of Medical Sciences of Ukraine, Ukraine. The purpose of the study was explained to the parents and patients before consent was obtained for their voluntary participation. Children 14–18 years of age consented together with their parents.

## 3 RESULTS

Table 1 shows the clinical and demographic characteristics of 213 patients who completed questionnaires (131 by proxy by parents), excluding those who were 18 years or older. Notably, only 15.5% did not screen positive for symptoms of anxiety as scored by the GAD‐7. Using the PHQ‐9, 54.9% screened positive for symptoms of depression.

**TABLE 1:** Demographic and clinical characteristics for the entire population and comparing those below and above HTQ scores indicating high risk for PTSD.

	HTQ ≤2.5	HTQ >2.5	Total	*p*‐value
*N*, *n* (%)	182 (85.5)	31 (14.6)	213 (100)	
Age, mean (SD)	8.65 (4.29)	7.40 (4.10)	8.47 (4.28)	.14
Sex Female, *n* (%)	80 (44)	20 (64.5)	100 (47)	.03
Epilepsy duration, m (SD)	5.49 (4.07)	5.30 (3.90)	5.46 (4.04)	.80
Seizures year before war, *n* (%)	135 (74.2)	22 (71)	157 (73.7)	.71
Seizures increase after war, *n* (%)	97 (53.3)	19 (61.3)	116 (54.5)	.41
Changed residence, *n* (%)	82 (45.1)	16 (51.6)	98 (46)	.5
Residence outside, *n* (%)	30 (38.5)	6 (37.5)	36 (38.3)	.94
Difficulty getting medical care, *n* (%)	89 (48.9)	20 (64.5)	109 (51.2)	.11
Difficulty getting ASMs, *n* (%)	138 (75.8)	23 (74.2)	161 (75.6)	.85
GASE score, m (SD)	3.53 (1.60)	4.81 (1.62)	3.71 (1.66)	<.001*
GASE categories, *n* (%)				
Low Severity (<3)	59 (32.4)	3 (9.7)	62 (29.1)	.01
Medium Severity (3–5)	97 (53.3)	18 (58.1)	115 (54)	
High Severity (>5)	26 (14.3)	10 (32.3)	36 (16.9)	
Total number of ASMs, *n* (%)				
0	20 (11)	3 (9.7)	23 (10.8)	1.00
1	59 (32.4)	10 (32.3)	69 (32.4)	
2	55 (30.2)	9 (29)	64.00 (30.1)	
3	38 (20.9)	7 (22.6)	45 (21.1)	
4	9 (5)	2 (6.5)	11 (5.2)	
5	1 (.6%)	0 (0%)	1 (.5)	
GAD‐7 score, m (SD)	8.95 (4.75)	15.71 (4.10)	9.93 (5.23)	<.001*
GAD‐7 levels of anxiety, *n* (%)				
None (<4)	33 (18.1)	0 (0)	33 (15.5)	<.001*
Mild anxiety (5–9)	66 (36.3)	3 (9.7)	69 (32.4)	
Moderate anxiety (10–14)	61 (33.5)	3 (9.7)	64 (30.1)	
Severe anxiety (>14)	22 (12.1)	25 (80.7)	47 (22.1)	
PHQ‐9 score, m (SD)	9.41 (4.81)	18.71 (5.25)	10.76 (5.87)	<.001*
PHQ‐9 cutoff, *n* (%)				
<10	94 (51.7)	2 (6.5)	96 (45.1)	<.001*
≥10	88 (48.4)	29 (93.6)	117 (54.9)	
HTQ‐40 score, m (SD)	1.75 (.38)	2.98 (.29)	1.92 (.57)	<.001*

**Abbreviations:** ASMs, Antiseizure medicaitons; GASE, Global Assessment of Severity of Epilepsy; HTQ, Harvard Trauma Questionnaire; m, mean; SD, standard deviation.

* Statistically significant after Bonferroni correction.

We divided patients into those above and below a score of 2.5 in the HTQ, a marker of PTSD (Table 1). The age range of children in the study was 1–18 years, mean 8.48 ±4.28 years; 61 were over the age of 11 years. There were 113 boys (52%) and 100 girls (47%), and 31 (14%) screened positive for PTSD in the HTQ, of whom a significantly larger proportion were girls (64.5% vs 35.5%). Ninety‐eight children (46%) had changed residence since the start of the war, and of these, one third had moved outside the country. The mean duration of epilepsy was 3.71 years; 119 (56%) experienced an increase in seizure frequency after the beginning of the war, 161 (76%) endorsed difficulties obtaining ASMs, and 109 (51%) described difficulties accessing medical care. Children categorized as likely to have PTSD (HTQ >2.5), had statistically significantly worse self‐rated severity of epilepsy (GASE 3.5 vs 4.8), as well as indicators of anxiety (GAD‐7 15.71 vs 8.95) and depression (PHQ‐9 18.71 vs 9.41).

Acknowledging that the tools used have not been validated in younger children, we conducted a sensitivity analysis to assess differences in variables between <11 and >11 y. No significant differences were found for any variable in any of the tools used according to age, and no variable contributed significantly differently.

Eleven percent of patients were not taking ASMs, 32% were on monotherapy, and 57% were on polytherapy. The most frequently used ASMs were valproate, levetiracetam, vigabatrin, lamotrigine, topiramate, and clobazam (>10% of the population). The odds of facing difficulties accessing ASMs varied by type of ASM (Table 2) and were 10.5 times higher for topiramate, 7.8 times higher for oxcarbazepine (near significant, OR 7.8), 5.6 times higher for carbamazepine, 5 times higher for vigabatrin, and 2.3 times higher for levetiracetam.

**TABLE 2:** Logistic regression model predicting difficulties accessing ASMs, by type of ASM.

ASM	*N* (%)	OR	95% CI lower	95% CI upper	*p*‐value
Topiramate	24 (11.3)	10.45	1.26	86.84	.03
Oxcarbazepine	17 (8.0)	7.79	.90	67.09	.06
Ospolot	12 (5.6)	5.93	.68	51.54	.11
Carbamazepine	16 (7.5)	5.62	1.14	27.72	.03
Vigabatrin	36 (16.9)	5.04	1.59	16.00	.01
Clobazam	24 (11.3)	4.49	.67	30.07	.12
Zonisamide	6 (2.8)	4.01	.36	45.02	.26
Clonazepam	20 (9.4)	2.56	.49	13.47	.27
Levetiracetam	71 (33.3)	2.30	1.02	5.16	.04
Phenobarbital	5 (2.4)	1.76	.15	20.99	.66
Valproate	86 (40.4)	1.65	.76	3.60	.20
Lacosamide	3 (1.4)	1.56	.10	25.00	.76
Lamotrigine	32 (15)	1.00	.38	2.68	.03

*Note*: ORs indicate the odds of reporting difficulties obtaining an ASM, compared to not being on that ASM, holding all other ASMs constant. For example, the odds are 10.45 times higher that youth taking topiramate will have difficulties accessing ASM than those not taking topiramate. No patients on ethosuximide (*n* = 16) reported difficulties accessing ASMs. Other ASMs were used by <3 respondents.

**FIGURE 1:**
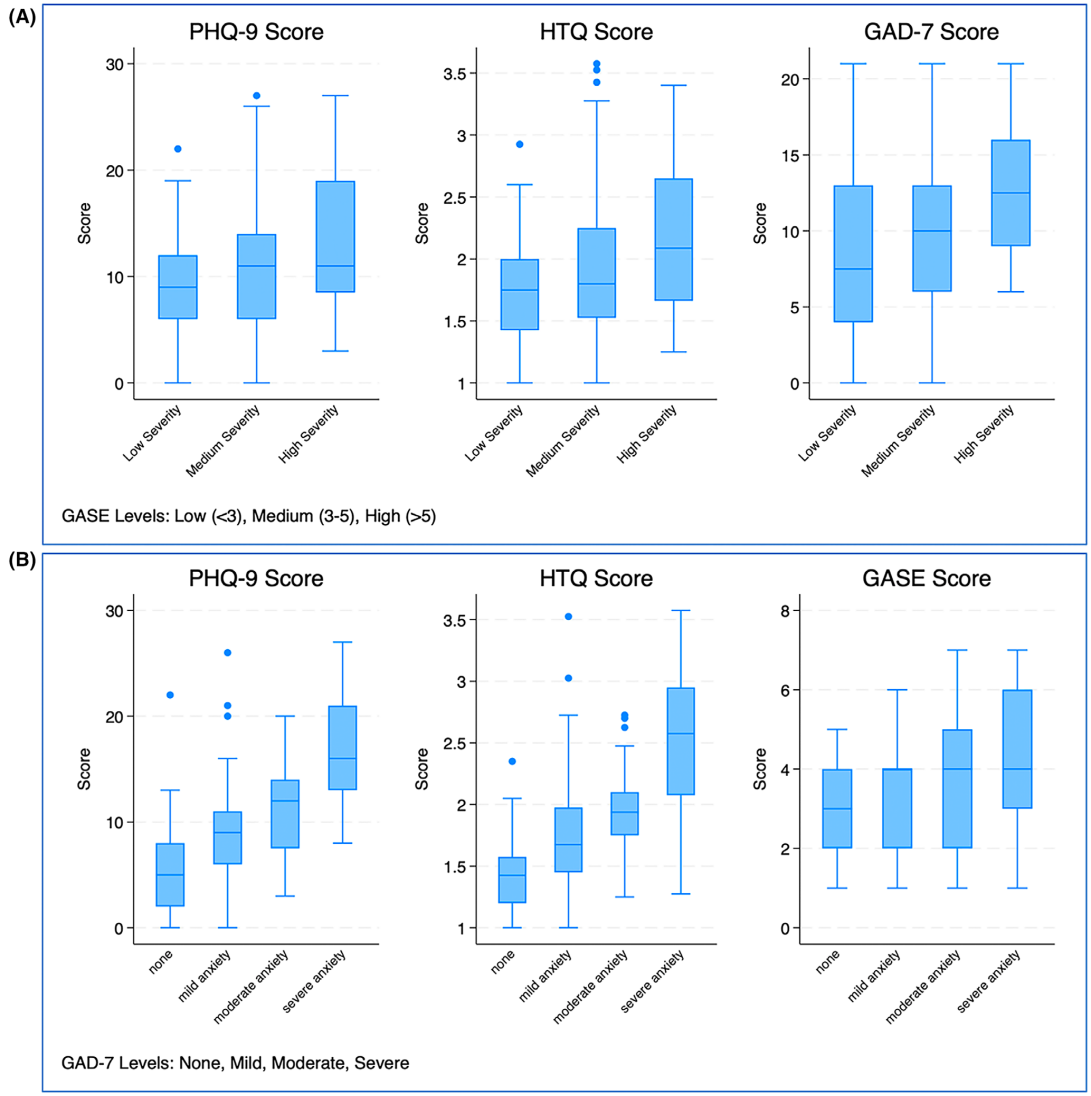
(A) Levels of severity of epilepsy and scores of depression, trauma symptoms, and anxiety. (B) Association between levels of anxiety symptoms, trauma scores, and epilepsy severity.

In order to categorize scores for analysis, we grouped the 7‐point GASE into low (1, 2), medium (3–5), and high (6, 7) categories, centering adjacent scale points around the midpoint (4). This approach mirrors the categories of other widely used 7‐point observational scales such as the Classroom Assessment Scoring System (CLASS), which interprets scores of 1–2 as low, 3–5 as intermediate, and 6–7 as high. Consistent with psychometric recommendations, adjacent categories were combined to yield three interpretable levels of severity while retaining ordinal information.^18^ Self‐reported severity of epilepsy (GASE) correlated with severity trauma scores (HTQ), depression (PHQ‐9), and anxiety (GAD‐7) in a graded fashion (Figure 1A). Similarly increasing levels of anxiety corresponded to increasing levels of depression (PHQ‐9) and trauma scores (HTQ), but less clearly to self‐reported epilepsy severity (GASE) (Figure 1B).

**FIGURE 2:**
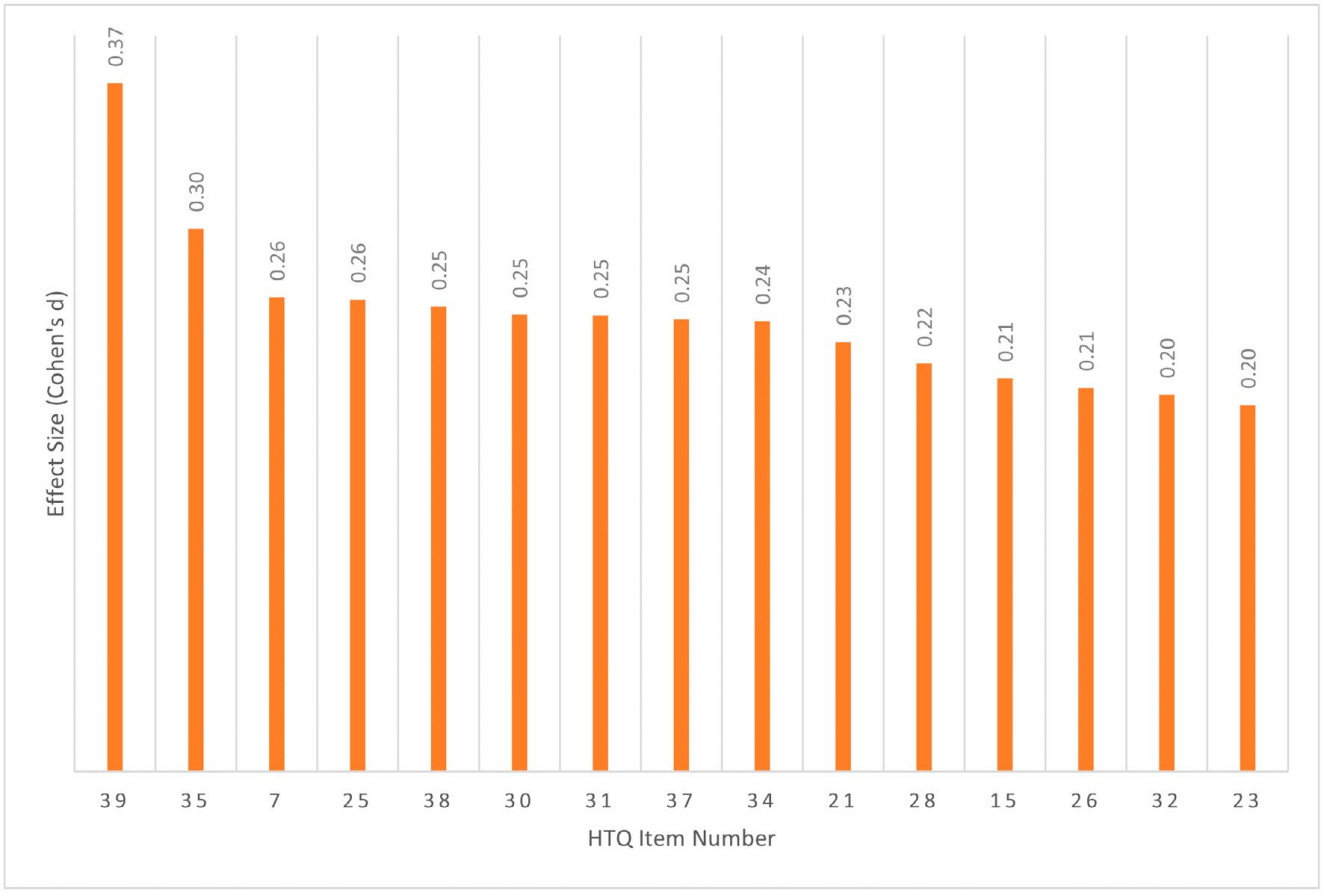
HTQ items with the largest difference between females and males (effect size Cohen's *d*). *item worse in males; all other items worse in females.

Research has shown that males and females respond differently to items in the HTQ, with females often endorsing higher levels of traumatic experience. Accordingly, we compared responses to individual HTQ items in females and males (Table S1). Two items were scored significantly higher by females: Item 35 (feeling humiliated) and Item 39 (feeling you are the only one who suffered). Many of the differences were small; however, 15 items demonstrated differences corresponding to small to medium effect sizes (Figure 2). Of these, only Items 7 (difficulty concentrating) and 15 (avoid thoughts/feelings of event) map to classic PTSD domains, whereas the rest refer to additional symptoms.

**Table:** 

#	Item
39	You are the only who suffered
35	Humiliated by event
7	Difficulty concentrating
25	Feel split into two people
38	Think why this happened to you
30	Ashamed of events
31	Others don't understand you
37	Unable to help others
34	Someone betrayed you
21	Physical symptoms
28	Guilty for surviving
15	Avoids thoughts/feelings of event
26	Unable to make plans
32	Others are hostile
23	Doing things you cannot remember

Multiple regression models of variables associated with severity of scores in depression and anxiety symptoms are shown in Table 3. Epilepsy severity was a significant predictor of depression, anxiety, and PTSD symptoms. Trouble accessing medical care was a predictor of more severe depression and anxiety scores. In addition, depression was also associated with trouble accessing medical care. Higher ratings of severity were predicted by younger age, difficulties accessing ASMs and medical care, and having increased seizures after the war. Higher levels of anxiety were predicted by younger age, trouble accessing medical care, and self‐rated epilepsy severity. Surprisingly, having seizures the year before the war (associated more severe epilepsy) predicted lower levels of anxiety. Further exploration through mediation analysis demonstrated a complex relationship, confounded by epilepsy severity (Figure S1). Therefore, this finding requires cautious interpretation.

**TABLE 3:** Multiple linear regression models of clinical and demographic variables associated with scores of GAD‐7, PHQ‐9, HTQ, and GASE scales.

	*B* coefficient	LCI_95_	UCI_95_	*p‐* value
**Predictors of GAD‐7 scores**				
Age	−.16	−.33	.00	.05
Seizures in year before war	−1.87	−3.53	−.20	.03
Trouble getting medical care	1.62	.01	3.23	.05
Epilepsy severity (GASE)	.76	.30	1.22	.001
**Predictors of PHQ‐9 scores**				
Trouble getting medical care	2.33	.50	4.16	.01
Epilepsy severity (GASE)	.72	.20	1.24	.007
**Predictors of HTQ scores**				
Epilepsy severity (GASE)	.09	.04	.14	<.001
**Predictors of GASE scores**				
Age	−.08	−.13	−.3	.001
Trouble getting ASMs	.78	.17	1.40	.01
Trouble getting ASMs plus trouble getting medical care	2.26	.71	3.80	.004
Seizures in year before war	1.05	.57	1.53	<.001
Increased seizures after war	.63	.21	1.05	.004

*Note*: All models used the same independent variables (see text for full list). Mental health instruments were strongly correlated with one another, therefore they were not utilized as predictors in the multivariate models (Figure S2).

## 4 DISCUSSION

In this study we have shown the impact of conflict on children and young people with epilepsy in Ukraine, utilizing a questionnaire based on standardized scales screening for symptoms of PTSD, anxiety, and depression, and assessing severity of epilepsy. As scored by the GAD‐7, 84.5% screened positive for symptoms of anxiety, and a relatively high proportion screened positive for symptoms of depression, with 54.9% scoring within the depressed range. These rates are high compared to other epilepsy populations assessed outside a conflict situation, with rates shown in school age children of 7% depression and 13% anxiety,^19^ albeit higher in adolescents (32.8% anxiety and 22% depression^20^). Armed conflicts are known to have a major impact on the mental health of affected populations, with PTSD and depression the most common mental disorders in the aftermath of war for both adults and children, reportedly occurring in up to one third of the people directly exposed to traumatic war experience.^21^ Therefore it is a little surprising that only 14% of children in our study scored within the PTSD range.

Almost twice the proportion with scores in the PTSD range were seen in girls than boys. It has been widely reported that females report higher levels of traumatic experience.^22^ Whereas two items were scored significantly higher by females, 15 were scored at small to medium effect sizes, but only two mapped to classic PTSD domains. Other items, for example, feeling humiliated and being unable to help others, could be closely linked to anxiety. Children categorized as likely to have PTSD (HTQ >2.5), had statistically significantly worse indicators of anxiety (GAD‐7 15.71 vs 8.95) as well as depression (PHQ‐9 18.71 vs 9.41) and self‐rated severity of epilepsy (GASE 3.5 vs 4.8).

As demonstrated previously in adults,^6^ the conflict appeared to have an impact on the epilepsy itself. Fifty‐six percent reported an increase in seizure frequency, 76% reported difficulty in obtaining medication, and 51% difficulties in obtaining medical care. The difficulties in accessing medication did not appear related to age, the type of ASM, or whether the ASM was licensed within country, although this may have had an influence on availability of some ASMs. A significant proportion (46%) also reported being displaced by the conflict, one third to outside the country. Parenting practices have been reported to play a crucial role in children's psychological well‐being in a war context, both as a risk and a protective factor.^21^ With a degree of proxy completion of questionnaires it is difficult to know how much of this has contributed to the results. Access to medical care at an individual and family level would appear crucial to minimizing the effect of conflict on those with epilepsy.^6^ It could be argued that many children have been protected from the conflict through displacement; however, multivariate analyses did not identify change of residence as a predictor (either positive or negative) of HTQ scores, anxiety, or depression.

Limitations of this questionnaire‐based study include that it could be completed by the child or by proxy. Several of the questionnaires used have not been been validated for completion by proxy. Furthermore, the drawback of proxy responses, seen in more than 50% cases, is the extent that answers are a reflection of the responders rather than the children themselves. Proxy‐completed questionnaires could be presumed to report more of the perceived child emotional and behavioral symptoms rather than being a direct assessment of internalizing psychopathology. Some studies have suggested cross‐informant agreement to be, on average, lower for internalizing symptoms such as depression and anxiety than for observable externalizing problems.^23^, ^24^, ^25^ However other studies, notably of displaced adolescent refugees have shown a close correlation between caseworker and self completion of questionnaires with regard to internalizing symptoms. Proxy underappreciation of emotional state could be particularly relevant to the HTQ scores, which could therefore give a falsely low value for PTSD. However, there are no data regarding the performance of the HTQ in children with proxy responses; it is designed for self report. That said, there is precedence for proxy use of the PHQ‐9 in adult glioma, with proxies reporting more depressive symptoms than patients, but being more reliable when reporting observable behavioral symptoms.^26^ In addition, instruments were used in our study (GAD‐7, PHQ‐9) outside the age range for which they are validated. In fact there are no instruments validated for children younger than 8 years of age. PHQ‐9 and GAD‐7 have been shown to perform poorly in screening for clinical depression and anxiety in children. When using the two instruments for diagnosis, 11 years of age has been found to be the optimal cutoff for both clinical depression and anxiety.^20^ We were restricted by the availability of instruments in Ukraine and at a time of conflict. This said, our population included a high proportion of adolescents. In addition, a sensitivity analysis revealed no significant difference in contribution of variables at <11 years vs >11 years of age. We acknowledge that these are significant limitations but wished to better understand the impact on war utilizing what we had available, with meaningful results still achieved.

This study has shown the highly complex relationship of severity of epilepsy, PTSD, and comorbid conditions. That said, trouble accessing medical care was a predictor for more severe depression and anxiety scores. In addition, depression was also associated with trouble accessing medical care. Enhancing access to medical care may be difficult at times of conflict, but such results can be utilized to highlight the need for ongoing support for individuals with epilepsy. A recent scoping policy highlighted that mental health and psychosocial interventions for war‐affected children should be multileveled, specifically targeted toward the child's needs, trauma‐informed, and strength‐ and resilience‐oriented.^27^ Trauma‐informed care is built around three important pillars: safety, connections, and managing emotions. It attempts to translate trauma research into practice to inform and improve care efforts, practically addressing trauma and promoting resilience through a person centered approach, taking into account what has happened rather than just what is wrong.^28^ Screening and assessment of the child's mental health burden and resources are indicated to inform targeted interventions.^2^ This is particularly relevant to those with epilepsy, and in girls, noting the higher prevalence of PTSD in this study.

## AUTHOR CONTRIBUTIONS

All authors contributed to the conceptualization and design of the study, and all reviewed and edited the manuscript. Volodymyr Kharytonov was responsible for distribution of the survey and data collection. Samuel Wiebe performed analyses. Volodymyr Kharytonov and J Helen Cross drafted the manuscript. All authors approved the final version of the manuscript.

## CONFLICT OF INTEREST STATEMENT

Volodymyr Kharytonov has no conflict of interest. J. Helen Cross has participated in clinical trials sponsored by Zogenix/UCB Pharma, GW Pharma/Jazz Pharmaceuticals, Stoke Therapeutics, Encoded, Epigenyx, and Ultragenyx, and sat on advisory boards for Nutricia, Biocodex, UCB, Stoke Therapeutics, and Takeda, unrelated to this work and all with remuneration to her department. Andriy Dubenko has no conflicts of interest. Samuel Wiebe has received unrestricted educational grants on behalf of his institution from UCB Pharma, Sunovion, Eisai, Jazz Pharma, Paladin Labs, and LivaNova, and as an advisory board member for Paladin Labs and Jazz Pharma for work unrelated to this project.

## ETHICS STATEMENT

We confirm that we have read the Journal's position on issues involved in ethical publication and affirm that this report is consistent with those guidelines.

## Supporting information


Appendix S1.

## Data Availability

The data that support the findings of this study are available from the corresponding author upon reasonable request.
