# Supplementary material for: Mental health of children with epilepsy in Ukraine during the war
Source: Epilepsia. 2026 Apr 21;67(7):3686–95. doi: 10.1002/epi.70251 (PMC13360895; doi:10.1002/epi.70251)

**Supplementary Table 1.**

**Pairwise comparison of individual HTQ items by sex, comparing mean scores by sex**

|  |  | Sex | | | |
| --- | --- | --- | --- | --- | --- |
|  |  | Female | Male | Total | p |
| # | Item | 100 (47) | 113 (53) | 213 (100%) |  |
| 1 | Thoughts of hurtful events | 2.02 (0.84) | 2.09 (0.92) | 2.06 (0.88) | 0.57 |
| 2 | Feel event happening again | 1.93 (0.90) | 1.96 (0.90) | 1.94 (0.90) | 0.84 |
| 3 | Recurrent nightmares | 1.80 (0.77) | 1.68 (0.75) | 1.74 (0.76) | 0.25 |
| 4 | Feel withdrawn | 1.95 (0.93) | 2.04 (0.93) | 2.00 (0.93) | 0.50 |
| 5 | Can't feel emotions | 1.64 (0.76) | 1.61 (0.78) | 1.62 (0.77) | 0.78 |
| 6 | Jumpy, nervous | 2.61 (0.87) | 2.58 (0.95) | 2.59 (0.91) | 0.78 |
| 7 | Difficulty concentrating | 2.24 (0.89) | 2.48 (0.96) | 2.37 (0.93) | 0.06 |
| 8 | Trouble sleeping | 2.49 (1.04) | 2.51 (0.95) | 2.50 (0.99) | 0.86 |
| 9 | Feel on guard | 2.37 (0.92) | 2.35 (0.97) | 2.36 (0.94) | 0.85 |
| 10 | Irritable, angry | 2.43 (0.84) | 2.39 (0.92) | 2.41 (0.88) | 0.74 |
| 11 | Avoid reminders of event | 1.89 (0.95) | 1.73 (0.78) | 1.80 (0.87) | 0.17 |
| 12 | Can't remember parts of event | 1.54 (0.76) | 1.46 (0.72) | 1.50 (0.74) | 0.43 |
| 13 | Low interest in activities | 2.18 (0.87) | 2.08 (0.81) | 2.13 (0.84) | 0.39 |
| 14 | Feels no future | 2.13 (1.03) | 1.95 (0.97) | 2.03 (1.00) | 0.18 |
| 15 | Avoids thoughts/feelings of event | 1.91 (0.95) | 1.72 (0.85) | 1.81 (0.90) | 0.12 |
| 16 | Sudden reaction when reminded of event | 1.96 (0.86) | 1.85 (0.93) | 1.90 (0.90) | 0.37 |
| 17 | Has fewer skills than before | 1.90 (1.02) | 1.74 (0.92) | 1.82 (0.97) | 0.24 |
| 18 | Difficulty coping with new | 2.03 (0.83) | 2.06 (0.89) | 2.05 (0.86) | 0.79 |
| 19 | Feel exhausted | 2.64 (0.99) | 2.55 (1.01) | 2.59 (1.00) | 0.51 |
| 20 | Body pain | 2.16 (1.03) | 2.04 (0.87) | 2.10 (0.95) | 0.38 |
| 21 | Physical symptoms | 2.20 (1.03) | 1.97 (0.90) | 2.08 (0.97) | 0.09 |
| 22 | Poor memory | 2.33 (0.95) | 2.27 (0.98) | 2.30 (0.97) | 0.63 |
| 23 | Doing things you cannot remember | 1.71 (0.89) | 1.55 (0.72) | 1.62 (0.81) | 0.15 |
| 24 | Difficulty concentrating | 2.23 (0.97) | 2.26 (1.00) | 2.24 (0.98) | 0.84 |
| 25 | Feel split into two people | 1.70 (0.90) | 1.48 (0.82) | 1.58 (0.87) | 0.06 |
| 26 | Unable to make plans | 2.05 (0.89) | 1.86 (0.94) | 1.95 (0.92) | 0.13 |
| 27 | Blaming self for events | 1.81 (0.93) | 1.68 (0.84) | 1.74 (0.88) | 0.29 |
| 28 | Guilty for surviving | 1.38 (0.78) | 1.22 (0.65) | 1.30 (0.71) | 0.11 |
| 29 | Hopelessness | 1.88 (1.04) | 1.69 (0.93) | 1.78 (0.98) | 0.16 |
| 30 | Ashamed of events | 1.59 (0.89) | 1.38 (0.79) | 1.48 (0.84) | 0.07 |
| 31 | Others don't understand you | 2.12 (1.14) | 1.86 (0.96) | 1.98 (1.05) | 0.07 |
| 32 | Others are hostile | 1.80 (0.93) | 1.62 (0.83) | 1.70 (0.88) | 0.14 |
| 33 | No one to rely on | 2.05 (1.10) | 1.88 (0.94) | 1.96 (1.02) | 0.24 |
| 34 | Someone betrayed you | 1.80 (1.01) | 1.58 (0.82) | 1.68 (0.92) | 0.07 |
| 35 | Humiliated by event | 1.63 (0.96) | 1.38 (0.71) | 1.50 (0.84) | 0.03 |
| 36 | Mistrust of others | 1.89 (0.95) | 1.81 (0.80) | 1.84 (0.87) | 0.49 |
| 37 | Unable to help others | 2.00 (1.04) | 1.77 (0.86) | 1.88 (0.95) | 0.08 |
| 38 | Think why this happened to you | 2.05 (1.02) | 1.80 (0.95) | 1.92 (0.99) | 0.07 |
| 39 | You are the only who suffered | 1.86 (1.02) | 1.50 (0.88) | 1.67 (0.96) | 0.01 |
| 40 | Need revenge | 1.49 (0.83) | 1.40 (0.83) | 1.44 (0.83) | 0.41 |

**Supplementary Figure 1. Mediation analysis to explore the interaction between seizures before the war and GASE score on the GAD7 scores**

**
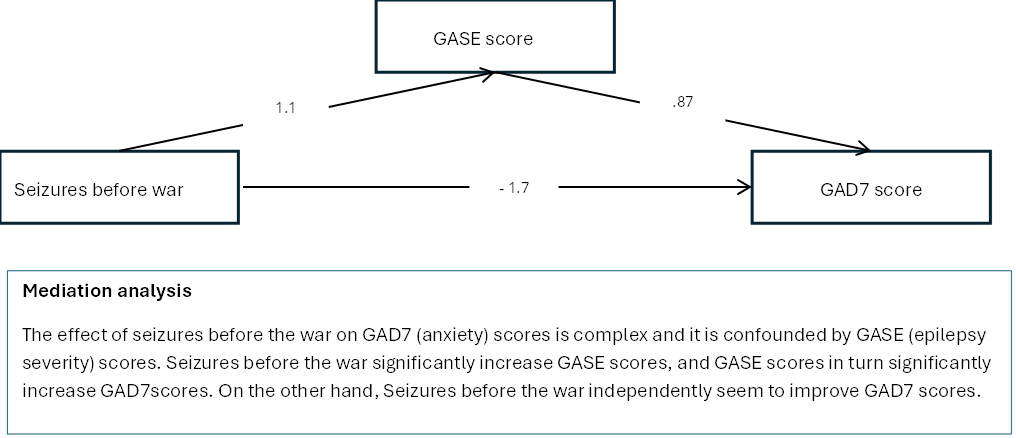
**

**Supplementary Figure 2. Heatmap of correlations among variables studied**


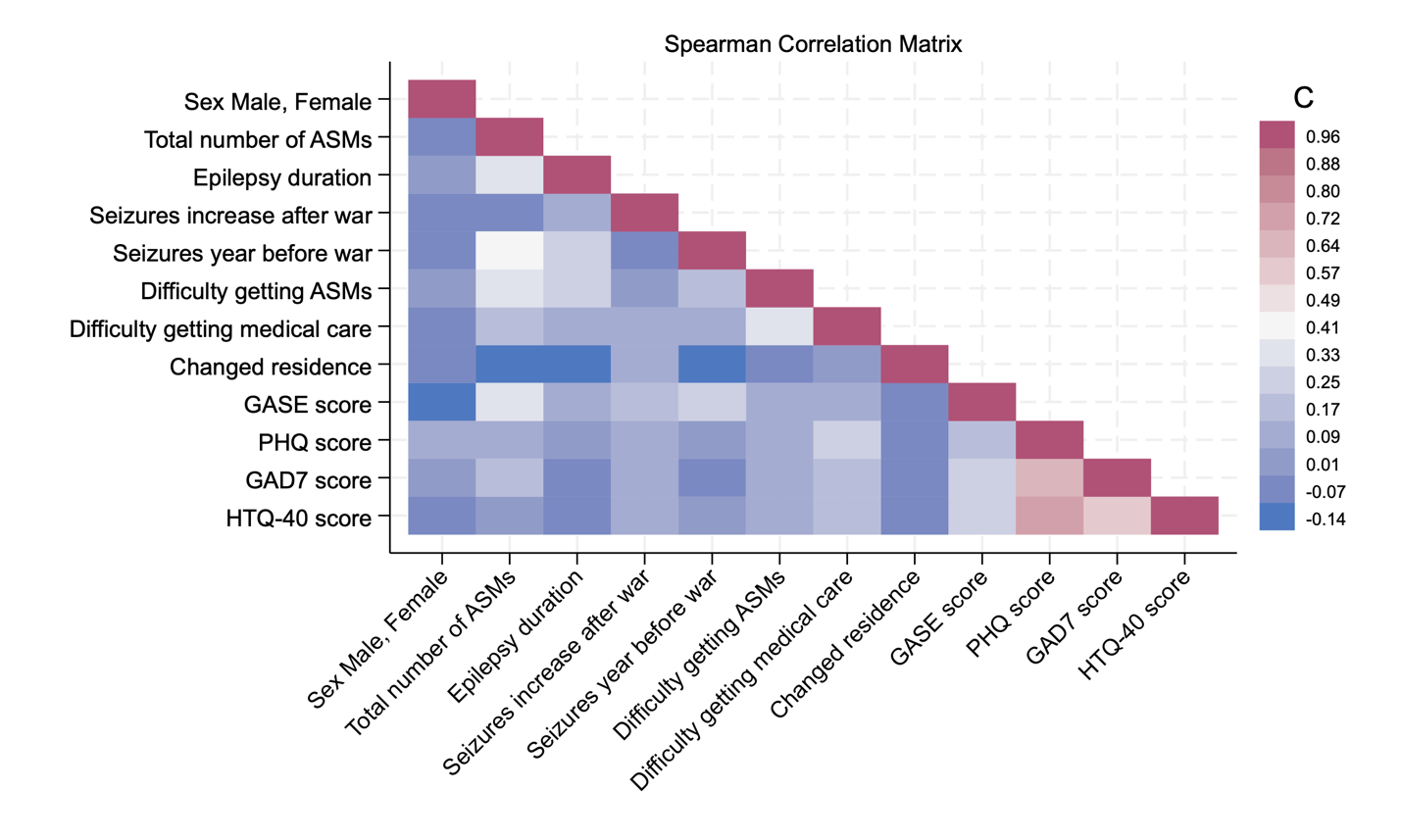

Supplement: Supplementary file 1 — Appendix S1. [file EPI-67-3686-s001.docx]
